# *ESR1* mutations are frequent in newly diagnosed metastatic and loco-regional recurrence of endocrine-treated breast cancer and carry worse prognosis

**DOI:** 10.1186/s13058-020-1246-5

**Published:** 2020-02-03

**Authors:** Adi Zundelevich, Maya Dadiani, Smadar Kahana-Edwin, Amit Itay, Tal Sella, Moran Gadot, Karen Cesarkas, Sarit Farage-Barhom, Efrat Glick Saar, Eran Eyal, Nitzan Kol, Anya Pavlovski, Nora Balint-Lahat, Daniela Dick-Necula, Iris Barshack, Bella Kaufman, Einav Nili Gal-Yam

**Affiliations:** 1 Cancer Research Center, Sheba Medical Center, Tel-Hashomer, Israel; 2 The Dr. Pinchas Borenstein Talpiot Medical Leadership Program, Chaim Sheba Medical Center, Ramat Gan, Israel; 3 Breast Oncology Institute, Sheba Medical Center, Tel-Hashomer, Israel; 4 NGS Unit, Cancer Research Center, Sheba Medical Center, Tel-Hashomer, Israel; 5 Bioinformatics Unit, Cancer Research Center, Sheba Medical Center, Tel-Hashomer, Israel; 6 Pathology Institute, Sheba Medical Center, Tel-Hashomer, Israel; 7 Sackler Faculty of Medicine, Tel Aviv University, Tel Aviv, Israel

**Keywords:** Breast cancer, *ESR1* mutation, Loco-regional/local recurrence, Metastasis, Endocrine treatment

## Abstract

**Background:**

Emerging mutations in the *ESR1* gene that encodes for the estrogen receptor (ER) are associated with resistance to endocrine therapy. *ESR1* mutations rarely exist in primary tumors (~ 1%) but are relatively common (10–50%) in metastatic, endocrine therapy-resistant cancers and are associated with a shorter progression-free survival. Little is known about the incidence and clinical implication of these mutations in early recurrence events, such as local recurrences or newly diagnosed metastatic disease.

**Methods:**

We collected 130 archival tumor samples from 103 breast cancer patients treated with endocrine therapy prior to their local/metastatic recurrence. The cohort consisted of 41 patients having at least 1 sample from local/loco-regional recurrence and 62 patients with metastatic disease (of whom 41 newly diagnosed and 28 with advanced disease). The 5 most common *ESR1* hotspot mutations (D538G, L536R, Y537S/N/C) were analyzed either by targeted sequencing or by droplet digital PCR. Progression-free survival (PFS), disease-free survival (DFS), and distant recurrence-free survival (DRFS) were statistically tested by Kaplan-Meier analysis.

**Results:**

The prevalence of *ESR1* mutations was 5/41 (12%) in newly diagnosed metastatic patients and 5/28 (18%) for advanced metastases, detected at allele frequency > 1%. All mutations in advanced metastases were detected in patients previously treated with both tamoxifen (TAM) and aromatase inhibitors (AI). However, in newly diagnosed metastatic patients, 4/5 mutations occurred in patients treated with TAM alone. PFS on AI treatment in metastatic patients was significantly shorter for *ESR1* mutation carriers (*p* = 0.017). In the local recurrence cohort, *ESR1* mutations were identified in 15/41 (36%) patients but only 4/41 (10%) were detected at allele frequency > 1%. Again, most mutations (3/4) were detected under TAM monotherapy. Notably, 1 patient developed *ESR1* mutation while on neoadjuvant endocrine therapy. DFS and DRFS were significantly shorter (*p* = 0.04 and *p* = 0.017, respectively) in patients that had *ESR1* mutations (> 1%) in their loco-regional recurrence tumor.

**Conclusions:**

Clinically relevant *ESR1* mutations are prevalent in newly diagnosed metastatic and local recurrence of endocrine-treated breast cancer. Since local recurrences are amenable to curative therapy, these mutations may inform the selection of subsequent endocrine therapies.

## Background

Hormone receptor-positive (HR+) breast cancers, expressing the estrogen and progesterone receptors, account for about 70% of all breast cancers [1]. Estrogen receptor (ER) is a transcription factor involved in cell proliferation and activation. Endocrine therapy is the mainstay of treatment in both local and metastatic HR+ tumors and includes ER inhibition by either ER modulators (i.e., tamoxifen (TAM)), ER degraders (i.e., fulvestrant), or estrogen deprivation by aromatase inhibitors (AI). Approximately 40% of patients diagnosed with local/loco-regional HR+ breast cancer treated with endocrine therapy will eventually develop recurrent disease [2, 3]. Recurrence events can be loco-regional metastases (about 3–8% of cases [4–6]), distant metastases, or both. The occurrence of loco-regional events increases the risk for the development of incurable distant recurrence and is associated with a poorer overall prognosis [7–9].

Among the various acquired endocrine resistance mechanisms, somatic mutations in the ER gene ligand-binding domain region (*ESR1*-LBD) had recently come under the spotlight through the advances of next-generation sequencing (NGS) technologies [10–13]. In vitro experiments have demonstrated that these *ESR1*-LBD mutations result in a ligand-independent constitutively activated ER, leading to proliferation and decreased sensitivity to endocrine treatments [10, 11, 13–16]. Since late 2013, there have been accumulating reports of *ESR1*-specific mutations leading to endocrine therapy resistance [10–13, 15, 17–29]. These mutations rarely exist (0–3%) in primary tumors [11, 15, 17, 24, 25] but are relatively common in metastatic endocrine therapy-resistant breast cancer lesions, with a wide-ranging prevalence of 6–55% [10–13, 15, 17–25, 27, 29]. The prevalence of *ESR1* mutations is governed by the sensitivity of detection methods as well as by the load of endocrine treatments prior to testing. Advanced digital droplet PCR (ddPCR) methods are more sensitive than NGS technologies [25, 26]. The ddPCR method combined with the high sensitivity to detect these mutations in cell-free DNA (cfDNA) isolated from the plasma [25, 30] clearly leverages the clinical implications of these mutations. A higher abundance of *ESR1* mutations has been described among metastatic patients treated with multiple lines of endocrine therapy (20–55%) as opposed to early metastatic patients (6–7%) [11, 18], supporting the theory that mutated clones are selected over treatment lines [31–33]. Additional studies are necessary to better understand the prevalence of *ESR1* mutations along the various stages of recurrent disease and their prognostic implications.

In this study, we aimed to describe the prevalence of *ESR1* mutations in early recurrence events, local recurrence, and newly diagnosed metastasis compared to heavily treated metastatic disease. We further investigated the associations with previous treatments and clinical outcomes in each subgroup. Our data demonstrate that *ESR1* mutations are present at newly diagnosed metastatic and local recurrence events and their presence is associated with survival. This work highlights the importance of testing *ESR1* mutations at the early stages of recurrence as this may determine the patients’ management including follow-up and changes in the treatment plan.

## Methods

### Breast cancer samples and clinical data

A retrospective cohort of hormone receptor-positive (HR+) breast cancer patients experiencing either local or metastatic recurrence was assembled based on available archival samples. All clinical data were obtained from the clinical records of the patients by an expert breast oncologist. This included age, TNM staging, grade, immunohistochemistry scores for estrogen receptor (ER), progesterone receptor (PR), human epidermal growth receptor 2 (HER2), and treatment lines. Outcome information included the date of next local/regional/metastatic recurrence, date of death, and date of the last follow-up. ER and PR positivity were determined based on local pathology practice (> 1% of positively stained cells). Archival formalin-fixed and paraffin-embedded (FFPE) tissue blocks were obtained from the Sheba Medical Center Pathology Institute. A total of 130 archival tumor samples were collected from 103 breast cancer patients who were treated with endocrine therapy prior to their recurrence and provided consent to sample and data collection. For 11 patients, matched samples from the primary tumor were available. In addition, for 14 patients, multiple samples from several recurrence events were collected. The cohort consisted of 41 patients having at least 1 sample from local (ipsilateral or contralateral breast) or regional (axillary nodes) recurrence and 62 patients with metastatic samples. The metastatic samples included 41 samples from newly diagnosed metastases and 28 samples from advanced metastatic disease. Samples from 1 normal breast reduction and 4 normal breast tissue adjacent to fibroadenomas served as controls. This project and tissue collection were approved by the institutional review board.

### DNA purification from FFPE samples

Tumor samples were sectioned and stained for hematoxylin and eosin to evaluate tumor cell percentage in the slide. Slides were reviewed by a pathologist to enrich for 70% cancer cellularity. One to 10 10 μM sections of the FFPE tumor samples were macro-dissected to enrich for tumor cellularity and deparaffinized at 90 °C for 5 min. Tumor DNA was isolated using the All Prep DNA/RNA FFPE Kit (QIAGEN) following the manufacturer’s instructions and stored at − 20 °C. DNA concentration was measured using the Qubit HS dsDNA kit (Invitrogen).

### *ESR1* amplicon sequencing

Hotspot mutations in the *ESR1* LBD region (527aa to 557aa) were identified by deep sequencing of a 129-bp amplicon at a coverage of about X10,000-X30,0000 using an Illumina platform. Ten nanograms of genomic DNA purified from FFPE samples was PCR amplified using the following primer: *ESR1* F: AACAAAGGCATGGAGCATCTG, *ESR1* R: CTCCACGGATGCCCCTC. The amplified PCR product was separated on and purified from a 2% agarose gel using a QIAGEN gel purification kit. For each sample, DNA concentration was measured by the Qubit HS dsDNA kit (Invitrogen). Library preparation steps were as follows: 50 ng of the PCR product was used for end repair and A-tail using NEB End prep enzyme mix (E7422s/E6090) for 20 min at 25 °C and 20 min at 72 °C. The DNA was purified using AMPure beads (Beckman Coulter), and ligation reaction was performed by T4 DNA quick ligase and index-oligo adaptor for Illumina platform for 15 min at 25 °C. The ligation product was cleaned again with AMPure beads and amplified by Illumina primers and hotstart DNA polymerase (Promega). The final library size was verified by TapeStation (Agilent). Paired-end sequencing (2 × 100 bp) was performed using Illumina HiSeq2500 or MiSeq (reagent micro kit, v2). Mutations were validated by either repeating library preparation and sequencing, or Droplet Digital™ PCR (ddPCR) (Bio-Rad).

### Sequencing data analysis

Sequence reads were mapped to reference human genome (hg19) using the NOVOALIGN. Five FFPE control samples (four normal breast adjacent to fibroadenomas and one normal breast reduction) were included in this study and were used for modeling statistical errors rates in each sequence read position. Under the assumption of a normal distribution for each sequence read position, only positions having non-reference allele frequency > 3*σ* (standard deviation) were reported as events. As 10 ng genomic DNA represents the content of ~ 1600 different diploid female human cells, we limited our sequence results to samples harboring ≥ 1% altered sequence reads.

### ddPCR for *ESR1* mutations detection

Bio-Rad QX100 Droplet Digital PCR (ddPCR) System was used for testing mutation abundance. Validation of the sequencing data was performed with primers and probes designed for D538G and wild type (WT). WT probe: CCCCTCTATGaCCTGCT-HEX, D538G mutant: CTCTATGgCCTGCTGC-FAM 900 nM for *ESR1* F primer: TACAGCATGAAGTGCAAG, and *ESR1* R primer: TGGGCGTCCAGCA were also used. The local recurrence cohort was examined using Bio-Rad-specific kits for D538G, L536R, and Y537S/N/C mutations. Digital PCR conditions were optimized with a temperature gradient to identify the optimal annealing/extension temperature on a QX200 ddPCR system (Bio-Rad) using TaqMan chemistry. Ten to 50 ng DNA from FFPE samples were used in each reaction, with the addition of 10 μl of ddPCR Supermix for probes (no dUTP) (Bio-Rad) and 250 nM TaqMan WT probe and 250 nM TaqMan D538G probe in a volume of 20 μl for each reaction. The emulsified PCR reaction was partitioned into ~ 14,000 droplets per sample in a QX100 droplet generator according to the manufacturer’s instructions.

### Quantitative ddPCR analysis

Plates were read on a Bio-Rad QX100 droplet reader using QuantaSoft v1.6.6.0320 software (Bio-Rad). At least two negative control wells with no DNA were included in every run and one positive sample for the mutant (using gBLocks from IDT of purified mutated FFPE DNA sample). A sample was considered mutation positive if it contained three or more mutant droplets. The detection limit was 0.1%. The results are reported as a fractional abundance of mutant DNA alleles to total (mutant plus WT) DNA alleles.

### Statistical analysis

*ESR1* mutation status was defined as a binary parameter (mutated at any AF vs. WT or AF > 1% vs. AF < 1% that includes WT). Two-tailed *p* values were considered significant if *p* < 0.05. The dissimilarity between the percentages of censored cases was tested by chi-square tests. The normal distribution of the clinical parameters was tested for by Kolmogorov-Smirnov test. To determine if there were differences between the groups (*ESR1* mutated vs. WT) in terms of the clinical parameters, we used the appropriate tests depending on the covariate type. An independent-samples *t* test was run for the normally distributed parameter (age). The non-parametric independent-samples Mann-Whitney test was used for clinical parameters that were not normally distributed (duration of endocrine treatment post-recurrence). The Fisher’s exact test was used for categorical covariates (stage, lymph node status, local recurrence type—local or regional). A chi-square test for the association was conducted between the mutation group and clinical parameters. Survival analyses were conducted by Kaplan-Meier survival analyses using the log rank (Mantel-Cox) test. Progression-free survival (PFS) was calculated as the time from AI treatment to any recurrence or death event. Disease-free survival (DFS) was calculated as the time from the tested local recurrence to any recurrence or death event. Distant recurrence-free survival (DRFS) was calculated as the time from either diagnosis or from the tested local recurrence to any distant recurrence or death event. Univariate Cox regression was used to calculate the hazard ratio for each parameter. Data analysis was performed using Statistical Package for the Social Sciences (SPSS), version 25 (IBM SPSS Statistics, Armonk, NY, USA) or with MATLAB® and Statistics Toolbox Release 2016b, The MathWorks, Inc., using the LogRank and KMPLOT functions by G. Cardillo.

## Results

We assembled 2 cohorts of recurrent breast cancer patients: a cohort of 62 patients with metastatic disease and a cohort of 41 breast cancer patients with local/loco-regional recurrence. All patients had ER-positive disease in both primary and recurrent lesions and were subjected to endocrine therapy prior to the recurrent tested lesions.

### Prevalence of *ESR1* mutations in metastatic recurrence samples and their clinical significance

**Fig. 1:**
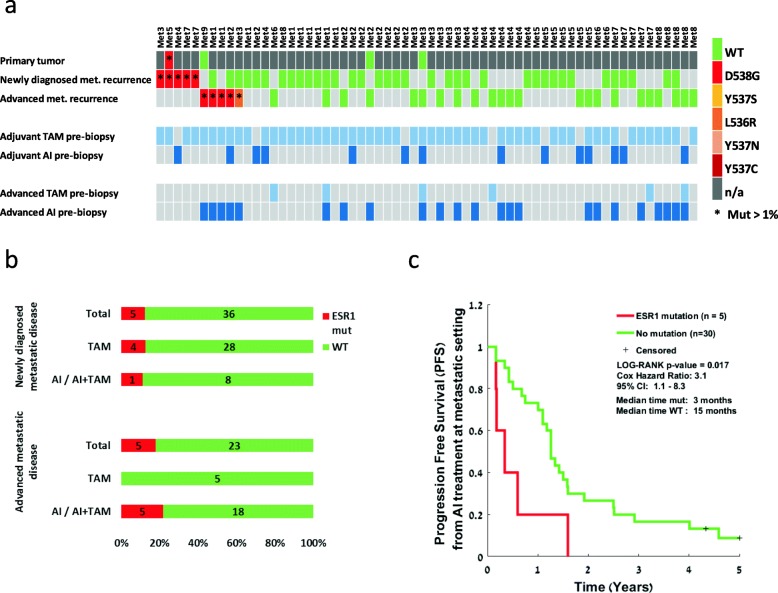
*ESR1* mutation analysis in the metastatic cohort and its clinical significance. **a** Analysis of matched samples from the metastatic cohort through the course of disease: primary tumor, newly diagnosed metastases, and advanced metastases. Samples are colored according to their mutation type. Red indicates *ESR1* Mut. Green indicates *ESR1* WT. *ESR1* mutations at an allele frequency of > 1% are marked by an asterisk. Dark gray indicates that a tumor was present at this time point but a sample was not available. Lower bars represent the treatments given for each patient pre-biopsy, either at the adjuvant phase before the metastatic disease or at the advanced phase before the advanced metastatic biopsy. TAM, tamoxifen, light blue; AI, aromatase inhibitor, blue. **b** Prevalence of *ESR1* mutations divided according to the metastatic disease stage and the type of treatment prior to biopsy. **c** Kaplan-Meier plots of progression-free survival calculated from the start of AI treatment at the metastatic setting

In total, we tested 41 FFPE samples from newly diagnosed metastases and 28 samples from advanced metastatic lesions (Fig. 1a) obtained from 62 patients. Prior endocrine treatments at each stage (either adjuvant or metastatic) are noted in the lower bars for each patient (Fig. 1a). For 4 patients, an additional sample from the primary tumor was available and tested. Patients’ characteristics are presented in Table 1.

**Table 1:** Clinicopathological characteristics

Metastatic recurrence cohort (*n* = 62)	*n* (%)
Age, mean (range)	46 (31–80)
Subtypes at primary diagnosis	
ER+/HER2−	38 (61%)
ER+/HER2+	13 (21%)
ER+/HER2 unknown	11 (18%)
T stage at primary diagnosis	
T0 - DCIS	2 (3%)
T1	12 (20%)
T2	33 (53%)
T3	7 (11%)
T4	3 (5%)
NA	5 (8%)
LN+ at primary diagnosis	
Negative	10 (16%)
Positive	44 (71%)
NA	8 (13%)
Chemotherapy for primary breast cancer	
Adjuvant	24 (38%)
Neoadjuvant	26 (42%)
Endocrine treatments	
Neoadjuvant	2 (3%)
Adjuvant TAM only	47 (76%)
Adjuvant AI/AI + TAM	14 (23%)
Advanced TAM only	3 (5%)
Advanced AI/AI + TAM	23 (37%)
Median follow-up from primary diagnosis, median years (range)	9.4 (3.3–27.7)
Median follow-up from metastasis, years (range)	3.9 (0.1–15.5)

*ESR1* hotspot mutations were found in 10/62 (16%) of the tested patients. Nine of 10 mutations were in D538G, and 1/10 mutation was in L536R. Mutations were detected in liver metastasis (4/10), bone metastasis (3/10), skin (1/10), mediastinum (1/10), and brain (1/10). To the best of our knowledge, *ESR1* mutations were not described before in brain metastasis [15]. To validate the sequencing results and allele frequencies, ddPCR analyses were performed on the mutated samples, resulting in a high concordance of allele frequencies (AF) of the mutated clones between both methods (Additional file 1: Table S1).

In line with previous findings [11, 18], *ESR1* mutation prevalence was lower among newly diagnosed metastases (5/41; 12%) and higher in advanced disease (5/28; 17%) (not significant by Fisher’s exact test) (Fig. 1b). Notably, 4/5 patients harboring *ESR1* mutations in newly diagnosed metastases (Met3, Met5, Met73, Met76) have been treated with TAM only as an adjuvant therapy without AI exposure. Mutated samples at advanced metastatic stages were detected after both TAM and AI treatment (Fig. 1b).

Matched primary tumors were available for four patients (Met5, Met9, Met24, Met34), two from *ESR1-*WT metastatic patients, and two from *ESR1*-mutated metastatic patients. The primary tumor of patient Met5 was positive for D538G mutation (AF of 22%, detected by ddPCR). The frequency of *ESR1* mutation in the metastatic sample was at AF of 39%, higher than in the primary tumor. Recurrence of metastatic disease for patient Met5 occurred early at 1.5 years post-surgery, while on TAM treatment.

To examine the prognostic significance of *ESR1* mutations, we performed a survival analysis and tested the interaction with other clinical parameters. No dissimilarity between the percentages of censored cases between the *ESR1* mutant and WT groups was found. No clinical parameters were significantly different between the groups (unpaired *t* test for age and chi-square test for other categorical parameters). DFS from the primary tumor to metastatic recurrence was similar for both mutant and WT cohorts. There was a trend for worse overall survival from the time of metastatic recurrence in the *ESR1* mutant group, which was not statistically different (Additional file 1: Figure S1), possibly due to the small sample size. However, PFS on AI treatment was significantly shorter in the *ESR1*-mutated patients, four of which were never previously treated with aromatase inhibitors (log rank *p* value = 0.017; median PFS 3 months for mutation carriers vs. 15 months for no mutation Cox hazard ratio 3.1 [95% confidence interval 1.1–8.3]) (Fig. 1c). These results are in line with previous observations that patients harboring *ESR1* mutations demonstrate lower progression survival rates under AI [34].

### *ESR1* mutations are prevalent in local recurrence and carry worse prognosis

**Fig. 2:**
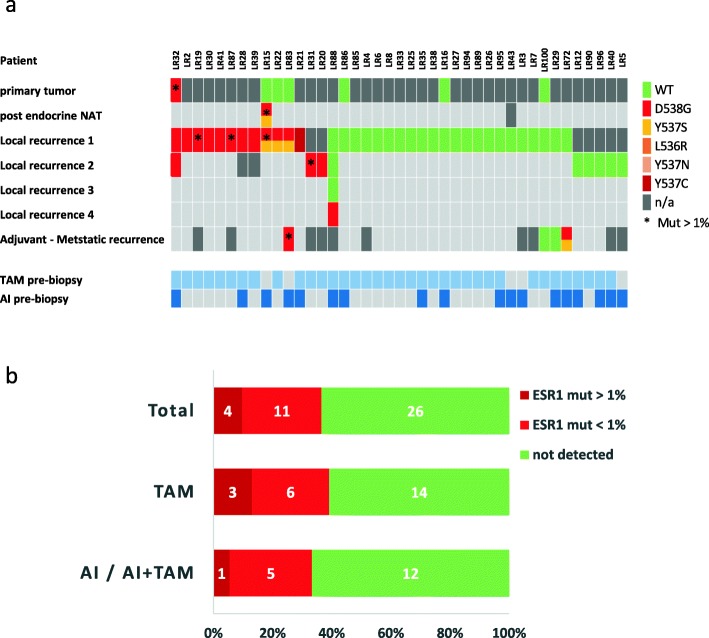
*ESR1* mutation analysis in the loco-regional cohort. **a** Analysis of matched samples from the loco-regional cohort through the course of the disease: primary tumor, all local recurrences, and advanced metastatic recurrence. Samples are colored according to their mutation type. Red indicates *ESR1* Mut. Green indicates *ESR1* WT. *ESR1* mutations at an allele frequency of > 1% are marked by an asterisk. Dark gray indicates that a tumor was present at this time point but a sample was not available. Lower bars represent the treatments given for each patient prior to the tested local recurrence sample. TAM, tamoxifen, light blue; AI, aromatase inhibitor, blue. **b** Prevalence of *ESR1* mutations in the loco-regional cohort divided according to the type of treatment prior to biopsy. *ESR1* mutations at an allele frequency of > 1% are colored with dark red

To test the prevalence of *ESR1* mutations, in the context of local recurrence, we assembled a cohort of 41 patients who experienced at least 1 local or loco-regional HR+ breast cancer recurrence (Fig. 2a). Patient characteristics are detailed in Table 2.

**Table 2:** Clinico-pathological characteristics

Local/regional recurrence cohort (*n* = 41)	*n* (%)
Age, median (range)	51 (27–84)
Subtypes	
ER+/HER2−	23 (56%)
ER+/HER2+	6 (15%)
ER+/HER2 unknown	12 (29%)
T stage at diagnosis	
T1	25 (61%)
T2	13 (32%)
T3	1 (2%)
T4	1 (2%)
LN+ at diagnosis	17 (41%)
Neoadjuvant endocrine treatment	2 (5%)
Endocrine treatments pre-biopsy	
Tamoxifen only	23 (56%)
Aromatase inhibitors (AI)	5 (12%)
Tamoxifen + AI	13 (32%)
Adjuvant/NAT chemotherapy	
Adjuvant	16 (39%)
Neoadjuvant	10 (24%)
Local/regional recurrence type	
Local recurrence	22 (54%)
Regional recurrence	19 (46%)
Unknown	1 (2%)
Distant recurrence	12 (29%)
Median follow-up time from diagnosis, years (range)	12 (4–24)
Median follow-up time from first local recurrence, years (range)	5 (0–20)
Median follow-up time from tested local recurrence, years (range)	4 (0–17)

All patients were treated in a curative intent for their local recurrence (surgery and if appropriate—radiation therapy). Additional primary tumors were available for 7 patients, and samples from a later metastatic recurrence were available for 4 patients. Two patients had samples from more than 1 loco-regional recurrence. One patient had undergone neoadjuvant endocrine treatment, and samples from pre- and post-treatment were available. Endocrine treatment before the first tested local recurrence biopsy is noted in the lower bars for each patient (Fig. 2a).

*ESR1* mutations were examined using hotspot-specific ddPCR assays performed on the FFPE samples. This method is more sensitive than NGS, with lower limits of detection of 0.05% [25, 26]. The detection limit in our analysis was set to 0.1%.

*ESR1* mutations were detected at any AF above the detection limit, in 15/41 (36%) patients with the D538G mutation being the most prevalent occurring in 14/15 patients. Y537C was detected in 1 patient while Y537S was detected together with D538G in 3 patients (Fig. 2a). *ESR1* mutations at local recurrence were mostly found in the first local recurrence. For 2 patients with *ESR1* mutation, only the second local recurrence was available. Interestingly, 1 patient had 4 available local recurrence samples, in which the first 3 were *ESR1*-WT and only the fourth recurrence harbored *ESR1* mutation.

*ESR1* mutated with AF greater than 1% were observed only in 4/41 (9.7%) patients. This cutoff was used for the sequencing data in the metastatic cohort. In line with the findings in the newly diagnosed metastatic cohort, mutations in the local recurrence samples were detected under TAM treatment alone, with no previous AI exposure. Mutations in TAM only-treated patients (23 patients) were found at any AF in 9/23 patients and at AF > 1% in 3/23 patients (Fig. 2b). There was no significant difference between patients developing mutations on TAM vs. AI/AI+TAM (Fisher’s exact test *p* = 0.75).

Matched primary tumors were available for seven patients in this cohort. One patient (LR32) harbored D538G mutation in the primary tumor, which reappeared in two local recurrences after 7 years and 16 years. Interestingly, the mutation AF in the primary tumor was 1.8% and remained at a similar level of AF (around 1% or less) in both local recurrences. This patient did not develop a metastatic disease, and her disease is stable for over 20 years.

Notably, patient LR15 was treated with endocrine neoadjuvant therapy (TAM for 2.5 months and AI for 18 months) after which she underwent definitive surgery. While no mutation was detected in the pre-treatment primary tumor, the post-treatment specimen harbored both D538G and Y537S mutations with high AF (35%). Although it is unlikely that with this high AF, the negative finding in the pre-treated primary tumor is due to sub-sampling, we cannot exclude the pre-existence of the mutation in very low AF. Following additional adjuvant AI therapy for 3.5 years, the patient had a loco-regional recurrence harboring the same mutations with similar AF. While the neoadjuvant treatment period was longer than the usual period (~ 6–12 months), this finding demonstrates that *ESR1* mutations can be selected upon neoadjuvant hormonal therapy.

To explore the prognostic and clinical implications of *ESR1* mutations in loco-regional recurrences, we performed survival analyses and tested the interaction with other clinical parameters. First, survival events were compared between patients with a mutation at any AF and without a mutation. In this case, the differences in survival distributions between the groups were not statistically significant (log rank test, *p* value > 0.05) (Additional file 1: Figure S2). There was no statistically significant difference (*p* value > 0.05) between the mutation groups and other clinical parameters (age at diagnosis, type of endocrine treatment, duration of endocrine treatment post-local recurrence, stage, LN, HER2 status, and type of local recurrence—local or regional). The differences between the groups were not significant also when adjusting for age and for the duration of AI post-local recurrence.

**Fig. 3:**
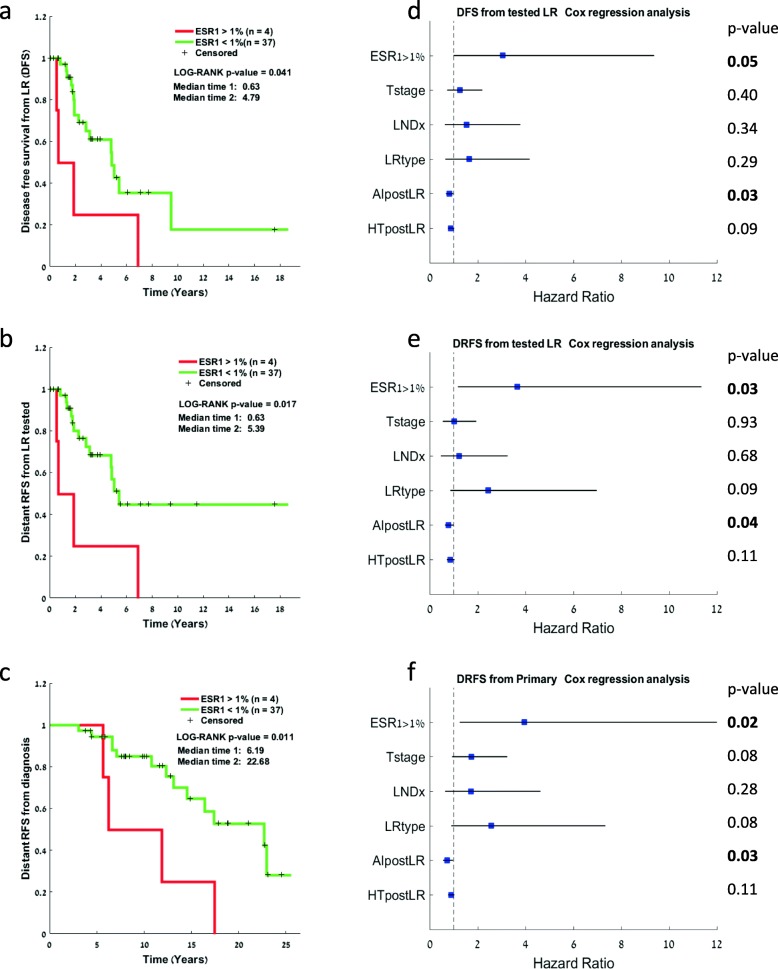
Survival analysis for patients with loco-regional recurrence. **a** Kaplan-Meier plots of distant recurrence-free survival from the primary tumor comparing *ESR1* mutations at an allele frequency higher versus lower than 1%. **b** Kaplan-Meier plots of disease-free survival from the tested local recurrence comparing *ESR1* mutations at an allele frequency higher versus lower than 1%. **c** Kaplan-Meier plots of distant recurrence-free survival from the tested local recurrence comparing *ESR1* mutations at an allele frequency higher versus lower than 1%. **d**–**f** Univariate Cox regression analysis for the same comparisons as in **a**–**c** represented by forest plots showing the hazard ratio for the various clinical parameters. LNDx, lymph node status at diagnosis; LR type, local or regional; error lines represent the 95% confidence interval

We speculated that *ESR1* mutation at low AF may not have a significant survival effect and therefore decided to test the effect on survival by setting a cutoff of 1% AF, similar to the cutoff used in the metastatic cohort. Kaplan-Meier survival analysis was conducted to compare the effect on survival between patients with mutations at first tested local recurrence with AF > 1% (*n* = 4 patients) vs. AF < 1% or no mutation (*n* = 37 patients). DFS from the first tested local recurrence was significantly shorter in the > 1% mutant-positive *ESR1* patients (log rank *p* value = 0.041, median time 0.63 [0–1.96] vs. 4.79 [2.59–6.77]) (Fig. 3a). Additionally, DRFS from tested local recurrence (Fig. 3b) as well as from primary tumor (Fig. 3c) was also significantly shorter in the AF > 1% *ESR1* mutant-positive patients (log rank *p* value = 0.017 and 0.011, respectively). A univariate Cox regression analysis that included other relevant clinicopathological features showed that besides *ESR1* mutations (AF > 1%), AI treatment post-local recurrence is significantly correlated with longer DFS and DRFS from local recurrence and from primary tumor diagnosis, respectively (Fig. 3d–f), while other parameters such as lymph node involvement at initial diagnosis trended towards worse outcomes but were not significant. Due to the small cohort size, we were unable to perform a multivariate analysis.

## Discussion

Activating *ESR1* mutations have been described in recent years, mainly in the context of endocrine-resistant metastatic breast cancer. Less is known regarding *ESR1* mutations status at the time of newly diagnosed HR+ breast cancer recurrence, specifically in non-metastatic loco-regional recurrence.

Here, we examined the prevalence and clinical significance of *ESR1*-LBD hotspot mutations in a retrospective cohort enriched for samples taken from either non-metastatic loco-regional recurrence treated with a curative intent or newly diagnosed metastatic recurrence prior to subsequent systemic therapy. All recurrent patients were exposed to adjuvant endocrine therapy prior to disease recurrence. To the best of our knowledge, this is the first description of *ESR1* mutations in a loco-regional breast cancer recurrence cohort.

We found *ESR1* hotspot mutation prevalence to be relatively high in our cohorts, both in newly diagnosed metastatic patients and loco-regional recurrent patients. Mutations’ frequency observed at the time of newly diagnosed metastatic disease recurrence post-adjuvant therapy alone was higher than previously described [18]. Furthermore, in both cohorts, we found a relatively high prevalence of ESR mutations developing in patients that received only TAM with no previous AI treatment: in 13/60 (22%) patients at any AF and in 7/60 (12%) at AF > 1%. Previous studies reported an ESR mutation prevalence in the range of 0–7% in metastatic patients without prior AI treatment [18, 35]. The higher incidence observed in our study relative to previous studies may be explained by the fact that our retrospective cohort was enriched for patients who were treated with adjuvant TAM only (median follow-up from diagnosis is 9.4 years [range 3.3–27.7]). Pre-existing *ESR1* mutations in the primary tumors, prior to exposure to any endocrine therapy, may also explain the high incidence of TAM selection in this cohort. *ESR1* mutations in the treatment-naive primary tumors were identified in two patients. In the metastatic patient, selection towards higher AF of *ESR1* mutation under TAM was observed. However, in the patient with local recurrence, despite the mutation being present in the primary tumor, no selection occurred, and the mutation remained at the same level of AF.

Nonetheless, our results clearly indicate that *ESR1* mutations can be detected after TAM treatment alone and are clinically relevant. Mechanistically, it was shown that *ESR1* mutations confer a pre-organized agonist state and an altered antagonist state which reduces SERM activity [36], thus providing the rationale for selective pressure for mutations emergence under TAM treatment.

Previous studies show that subsequent single-agent AI treatment in *ESR1* mutation metastatic carriers results in poor PFS [15, 18, 20, 21, 37]. Most patients in these studies were previously exposed to an AI. Here, we show that patients that have never been exposed to an AI and carry an *ESR1* mutation show poor PFS on single-agent AI. This supports the understanding that single-agent AI should not be used in patients with *ESR1* mutations even if they never received an AI. Combinations of AI or fulvestrant with CDK4/6 inhibitors are the current standard of care for first-line treatment of HR+ metastatic breast cancer. Prospective data are lacking for the efficacy of these combinations in *ESR1*-mutated metastatic patients. It has been shown that *ESR1* mutant cells retain their dependence on CDK4/6 and cyclin D; thus, CDK4/6 inhibitors seem to be active in *ESR1* mutant cells [36]. Since data from patients in the second-line setting (PALOMA-3, Bolero-2, SofEA trials) [20, 21, 29] provide evidence that fulvestrant is superior to AI in *ESR1*-mutated patients, it seems that fulvestrant combined with CDK4/6 will be more efficacious in these patients.

*ESR1* mutations in loco-regional recurrence were not previously systematically reported. We used ddPCR to analyze the samples from the loco-regional cohort. As ddPCR is more sensitive than NGS, it has the potential to detect low AF-mutated clones for which clinical relevance is unclear, especially in such a small cohort. The prevalence of *ESR1* mutations at all AF was high at loco-regional recurrences (36%) and may reflect a preponderance for *ESR1*-mutated tumors to recur locally. In these patients, more *ESR1* mutant pts. (40%) eventually progressed to metastatic disease compared to WT *ESR1* patients (30%), but this and other outcomes were not significantly different. However, in patients harboring mutations at AF > 1%, DFS and DFRS were significantly inferior compared to wild-type patients, possibly indicating a higher invasive/metastatic potential for mutation carrying tumors. In support of this, studies in mouse models have shown that cells harboring *ESR1* mutations had increased migratory capacity and that the D538G and Y537S mutants induce a unique E2-independent transcriptional program which promotes metastases [10, 38]. Thus, it may be speculated that recurrent tumors in which these mutations developed to a significant extent under the selective pressure of adjuvant endocrine treatment will have a higher recurrence/metastatic potential in the future.

The conclusions of this study may be limited by the relatively small cohort size. The prevalence and clinical significance of *ESR1* mutations in loco-regional recurrence should be validated in larger and possibly prospective cohorts. Furthermore, a standardized cutoff for mutational AF that is clinically significant should be set with the accumulation of additional studies that examine early and local recurrence events [39].

Validation of these results in larger cohorts may have major implications on the treatment algorithm in recurrent tumors. While the loco-regional recurrent cohort seemed to generally benefit from AI treatment (see our univariate analysis, Fig. 3), this may not be the optimal “adjuvant” treatment for the *ESR1* mutation carrying tumors. In these cases, agents such as fulvestrant, newer generation SERDs, or combinations with biologic agents such as CDK4/6 inhibitors may be preferable.

Finally, we provide evidence for the emergence of *ESR1* mutations under neoadjuvant treatment. In one patient, a high AF frequency D538G mutation developed during a period of neoadjuvant endocrine therapy and was later found again in the recurrent tumor in a similar frequency. Larger cohorts of pre- and post-neoadjuvant therapy-treated patients should be examined to understand if such mutations are indeed enriched under neoadjuvant treatment and what is their clinical significance. If found, these may also impact post-operative adjuvant treatment decisions.

## Conclusions

To summarize, the data presented here emphasize that clonal selection for hotspot *ESR1* mutations can occur at the early stages of both metastatic and local recurrence. These mutations can emerge after or during adjuvant endocrine therapy including single-agent TAM, as well as during neoadjuvant endocrine treatment of primary tumors. The occurrence of these mutations at least in AF > 1% confers a worse prognosis. These results have important implications for the future routine follow-up of patients to improve treatment plans and cancer outcomes. Further studies in the early recurrent setting will inform us of the optimal therapy in these patients.

## Supplementary information

**Additional file 1: Table S1.** validation of Sequencing results by ddPCR. **Figure S1.** DFS and OS for the metastatic cohort. Kaplan–Meier plots of a. disease free survival (DFS) from the primary tumor and b. overall survival (OS) from tested metastasis, comparing patients with *ESR1* mutations vs. no detected mutation at the metastatic sample. **Figure S2.** RFS, DFS and DRFS for the loco-regional cohort: mutant vs. WT. Kaplan–Meier plots of a. recurrence free survival (RFS) from the primary tumor, b. disease free survival (DFS) from the tested local recurrence (LR), c. distant recurrence free survival (DRFS) from the primary tumor, and d. distant recurrence free survival (DRFS) from the tested local recurrence (LR), comparing patients with *ESR1* mutations at any allele frequency vs. no detected mutation.

## Data Availability

NGS data will be deposited in NCBI’s Sequence Read Archive (SRA) and are available through SRA accession number no. upon acceptance.

## Abbreviations

AF: Allele frequencies
AI: Aromatase inhibitors
ddPCR: Droplet digital polymerase chain reaction
DRFS: Distant recurrence-free survival
DSF: Disease-free survival
*ESR1*: Estrogen receptor 1
FFPE: Formalin-fixed paraffin-embedded
HR+: Hormone receptor-positive
MUT: Mutant
NAT: Neoadjuvant treatment
NGS: Next-generation sequencing
PFS: Progression-free survival
RFS: Recurrence-free survival
TAM: Tamoxifen
WT: Wild-type
