# Correction to: ESR1 mutations are frequent in newly diagnosed metastatic and loco-regional recurrence of endocrine-treated breast cancer and carry worse prognosis

**DOI:** 10.1186/s13058-020-01265-y

**Published:** 2020-03-12

**Authors:** Adi Zundelevich, Maya Dadiani, Smadar Kahana-Edwin, Amit Itay, Tal Sella, Moran Gadot, Karen Cesarkas, Sarit Farage-Barhom, Efrat Glick Saar, Eran Eyal, Nitzan Kol, Anya Pavlovski, Nora Balint-Lahat, Daniela Dick-Necula, Iris Barshack, Bella Kaufman, Einav Nili Gal-Yam

**Affiliations:** 1 Cancer Research Center, Sheba Medical Center, Tel-Hashomer, Israel; 2 The Dr. Pinchas Borenstein Talpiot Medical Leadership Program, Chaim Sheba Medical Center, Ramat Gan, Israel; 3 Breast Oncology Institute, Sheba Medical Center, Tel-Hashomer, Israel; 4 NGS Unit, Cancer Research Center, Sheba Medical Center, Tel-Hashomer, Israel; 5 Bioinformatics Unit, Cancer Research Center, Sheba Medical Center, Tel-Hashomer, Israel; 6 Pathology Institute, Sheba Medical Center, Tel-Hashomer, Israel; 7 Sackler Faculty of Medicine, Tel Aviv University, Tel Aviv, Israel

**Correction to: Breast Cancer Res**

**https://doi.org/10.1186/s13058-020-1246-5**

After the publication of the original article [1], we were notified the upper panel of the Fig. 1, where the patients’ codes are listed, was cropped by mistake so the patients 1–8 are repeated. The 88 patients should be listed, instead.

Below is the correct version of Fig. 1.

**Fig. 1:**
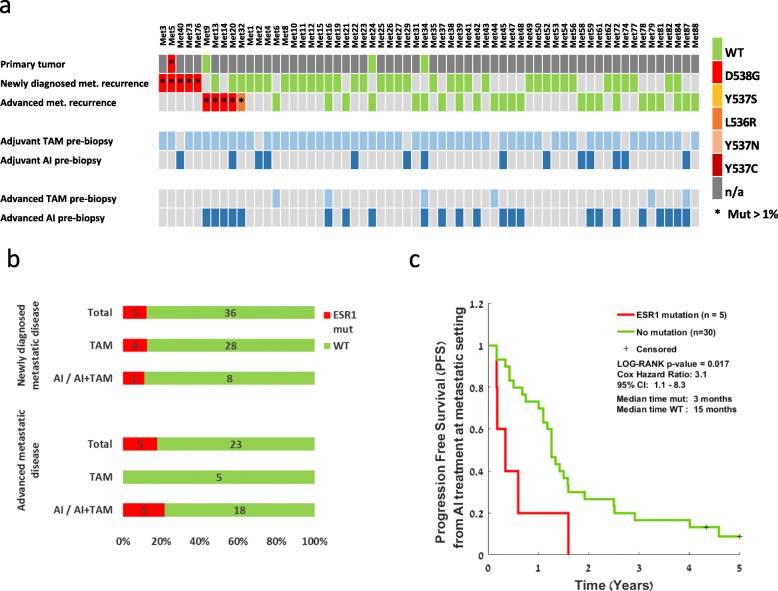
*ESR1* mutation analysis in the metastatic cohort and its clinical significance. **a** Analysis of matched samples from the metastatic cohort through the course of disease: primary tumor, newly diagnosed metastases, and advanced metastases. Samples are colored according to their mutation type. Red indicates *ESR1* Mut. Green indicates *ESR1* WT. *ESR1* mutations at an allele frequency of > 1% are marked by an asterisk. Dark gray indicates that a tumor was present at this time point but a sample was not available. Lower bars represent the treatments given for each patient pre-biopsy, either at the adjuvant phase before the metastatic disease or at the advanced phase before the advanced metastatic biopsy. TAM, tamoxifen, light blue; AI, aromatase inhibitor, blue. **b** Prevalence of *ESR1* mutations divided according to the metastatic disease stage and the type of treatment prior to biopsy. **c** Kaplan-Meier plots of progression-free survival calculated from the start of AI treatment at the metastatic setting
