# Supplementary material for: ESR1 mutations are frequent in newly diagnosed metastatic and loco-regional recurrence of endocrine-treated breast cancer and carry worse prognosis
Source: Breast Cancer Res. 2020 Feb 3;22:16. doi: 10.1186/s13058-020-1246-5 (PMC6998824; doi:10.1186/s13058-020-1246-5)

Supplementary Table S1:
validation of Sequencing results by ddPCR.

Sample ID	sequencing		validation: ddPCR	
	position	% reads	position	% fractional abundance
ESR 3	D538G	6.86	D538G	3.36
ESR 5	D538G	39.30	D538G	37.21
ESR 9	D538G	7.93	D538G	6.71
ESR 13	D538G	1.97	D538G	4.96
ESR 14	D538G	45.57	D538G	44.09
ESR 20b	D538G	37.93	D538G	37.97
ESR 40	D538G	42.74	D538G	41.94
ESR 73	D538G	1.01	D538G	10.93
ESR 6	D538G	not detected	D538G	not detected
ESR 20a	D538G	not detected	D538G	not detected
ESR 50	D538G	not detected	D538G	not detected
ESR 71	D538G	not detected	D538G	not detected

Figure S1:

DFS and OS for the metastatic cohort

A

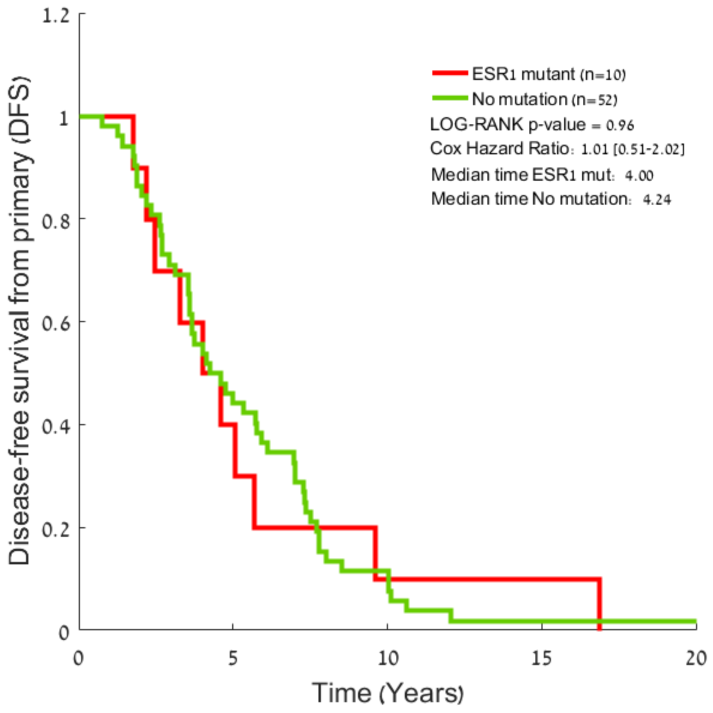

B

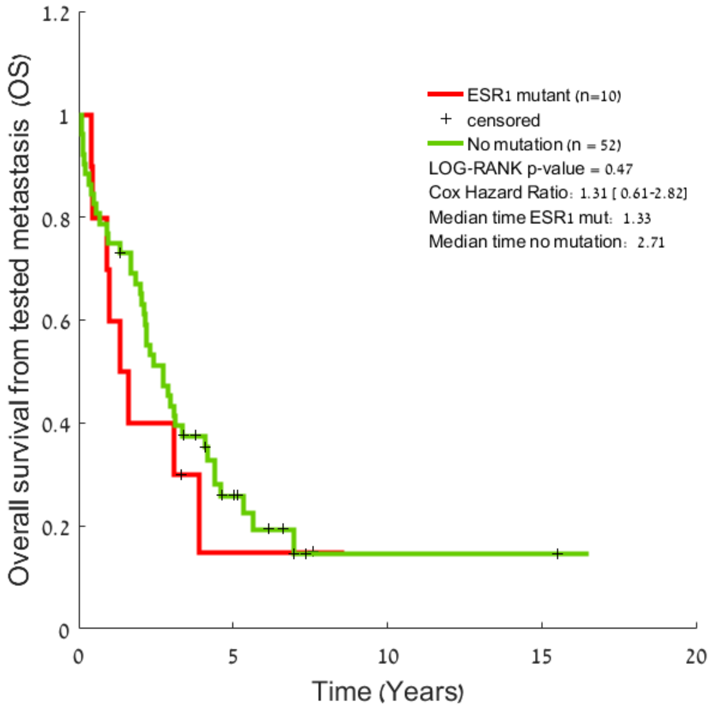

Figure S2:

RFS, DFS and DRFS for the loco-regional cohort: mutant vs. WT

A

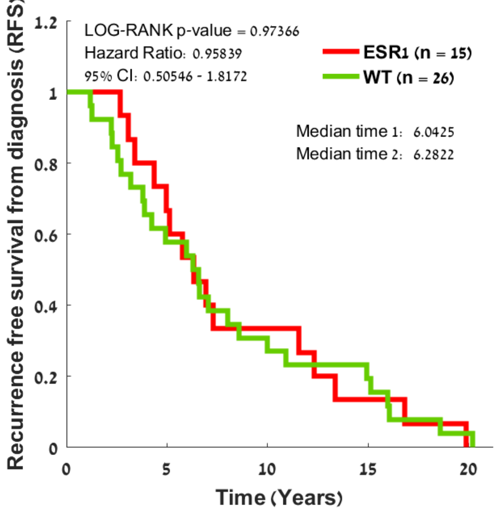

B

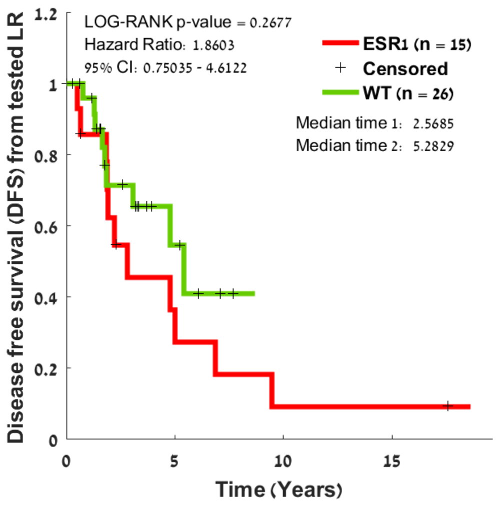

C

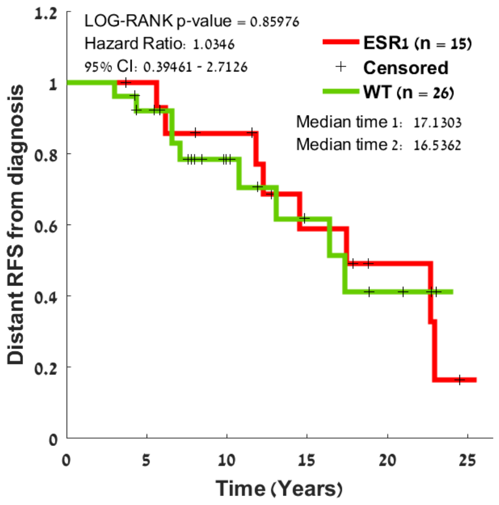

D

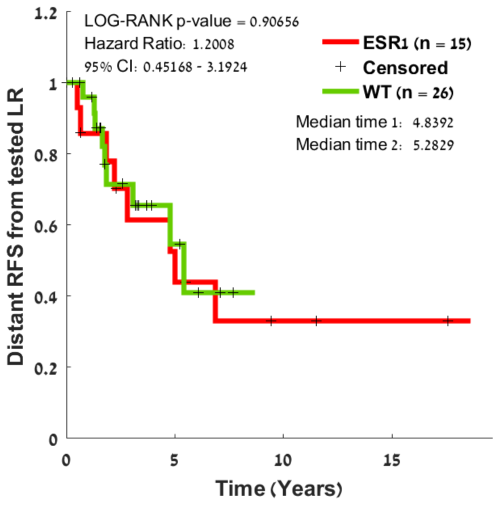

Supplement: Supplementary file 1 — Additional file 1: Table S1. validation of Sequencing results by ddPCR. Figure S1. DFS and OS for the metastatic cohort. Kaplan–Meier plots of a. disease free survival (DFS) from the primary tumor and b. overall survival (OS) from tested metastasis, comparing patients with ESR1 mutations vs. no detected mutation at the metastatic sample. Figure S2. RFS, DFS and DRFS for the loco-regional cohort: mutant vs. WT. Kaplan–Meier plots of a. recurrence free survival (RFS) from the primary tumor, b. disease free survival (DFS) from the tested local recurrence (LR), c. distant recurrence free survival (DRFS) from the primary tumor, and d. distant recurrence free survival (DRFS) from the tested local recurrence (LR), comparing patients with ESR1 mutations at any allele frequency vs. no detected mutation. [file 13058_2020_1246_MOESM1_ESM.pdf]
